# Mr. Tomes' Inaugural Address

**Published:** 1894-08

**Authors:** 


AMERICAN JOURNAL

—OF—

DENTAL SCIENCE.

Vol. xxviii. Baltimore, August, 1894. No. 4.

ARTICLE I.

### MR. TOMES' INAUGURAL ADDRESS.

The following extracts from the Presidental address of Mr. Tomes, at the annual meeting of the British Dental Association, in Newcastle, will be read with interest:

"It seems to me that I cannot better employ the short time which I propose to occupy, than by a short review of the conditions of a dentist's life—of that which conduces to his success or failure, and of the manner in which these conditions react upon the man himself; and in the fulfillment of this task I hope that I shall not be thought to play the part of a too candid friend.

"As a united body—and of this union this Association is the visible sign—we are very young; counted in the years of a man's life we have not attained to our majority, and what are twenty-one years in the history of a profession? and of youth no one can expect more than a promise. And if we venture to think that we show some promise, I also fear that we have many of the faults of youth—faults possesed by youth, however well endowed, faults that pertain to young corporations, and to young nations no less than to individuals. But though there may be excuse for our faults, that is not the less reason that we should try to recognize them, and so far as may be, correct them. It is in my mind that we expect too much, that we hope to go too fast, and that we are inclined to clamor for a degree of consideration which can only be accorded in the fulness of time, if ever. This consideration may take many forms; it may be more social recognition, it may be a higher scientific status, or it may be the confidence of the Legislature in entrusting us with more power to work out our ideas. But whatever form it is to take, it, in the very nature of things, can only be of slow growth, and by clamoring for it before it is accorded, we run the risk that is incurred by the pushing youth, of being snubbed for our pains.

"This aspect is not confined to our own specialty, it has seldom been better expressed than in the words of Dr. Mitchell Banks, so well known here in the north, and I will read you an extract from his address given last year before the Medical Society of London. Speaking of various medical organizations, he said: 'To become a gigantic mutual admiration body is a mistake. There can be nothing worse than to be ignorant of our weak places, and the man who, like the late Dr. Milner Fothergill, points them out to us, is certain to be a thousand times more alive to the real dignity of our profession than the vulgar persons who boast so much about it and add so little to it. By mere virtue of our profession we do not rank socially with other professions—we have to make our social position for ourselves. So much the more reason why our whole profession, down to its youngest graduate, should be men of such good general culture that their company should be welcomed not merely by the rich (for of these I make little account), but by all of those whose well-trained minds, whose liberal ideas, and whose refined manners, constitute the true society of our country.'

"So I shall not say much of the great strides that have been made, in the education gone through, in the standard of our professional examinations (our students have to pass the same preliminary examination in general education as the general medical students), nor of the progress which legislation has rendered possible in the hindering of irregular forms of practice—this has all been said before, *usque ad nauseam*; but will pass at once to point out the conditions which it appears to me are called for to make the successful practitioner. It goes without saying that he must have fully availed himself of his opportunities of study, for which there is now no lack of opportunity, and it would lead me too far afield to discuss the details of that training of hand and brain, but I should like to say a few words on the matter of a training beyond the ordinary routine of dental education; for there is a danger lest, led away by the pride of manipulative dexterity, we underrate directions of study which, to the thoughtless, seem to have little practical outcome.

"We have all of us made acquaintance with the self-styled practical man in all grades of society, from the artisan who poisons us with sewer gas, to the politician whose horizon is bounded by the limits of his personal observation, and that none too accurate. Let us quote to you the words of one of the clearest thinkers of our day, Professor Huxley, who thus delivered himself upon the proper scope of education: 'I often wish that this phrase, applied science, had never been invented, for it suggests that there is a sort of scientific knowledge of direct practical use, which can be studied apart from another sort of scientific knowledge, which is of no practical utility, and which is termed pure science. But there is no greater fallacy than this. What people call applied science is nothing but the application of pure science to particular classes of problems. It consists of deductions from those general principles, established by reasoning and observation, which constitute pure science. No one can safely make these deductions until he has a firm grasp of the principles and he can obtain that grasp only by personal experience of the operations of observation and of reasoning on which ey are founded. Almost all the processes employed in the arts and the manufactures fall within the range either of physics or of chemistry. In order to improve them, one must thoroughly understand them; and no one has a chance of really understanding them unless he has obtained that mastery of principles and that habit of dealing with facts which is given by long-continued and well-directed purely scientific training in the laboratory.'

"I will not weaken these pregnant words by comment, save only to say that every word which I have quoted is applicable to the training of the dentist, but that as yet we are far behind such an ideal as there is propounded. That scientific habit of mind by which we observe correctly and draw conclusions legitimately is essential, but it is fortunately one which can, to a great extent at all events, be cultivated. But do not suppose that I would allow this wider mental culture to at all take the place of that patient acquisition of manipulative, and I may say, empirical skill. To once more quote Professor Huxley: 'Indeed, I am so narrow-minded myself, that if I had to choose between two physicians, one who did not know whether a whale is a fish or not, and could not tell gentian from ginger, but did understand the application of the institutes of medicine to his art, while the other, like Talleyrand's doctor "knew a little of everything, even a little physic," with all my love for breadth of culture, I should assuredly consult the latter.'

"But in real life we are not called upon to make this choice; the man who is greedy of learning in his own special line is rarely—I may say, never—content to be ill-informed outside it. But supposing our young aspirant to start fully equipped with such knowledge as the schools can give him, his success is not yet fully assured, and there are certain qualities, like all qualities capable of improvement by cultivation, which will serve him in good stead. He must have nerve; not perhaps the nerve of the surgeon in whose hands lie the issues of life and death, but a certain steadiness of nerve which will enable him in the face of his special difficulties to be fully master of all the skill which he possesses, and this will go far towards securing the confidence of his patients. He must be painstaking, for it is in attention to *minutiæ* that, just as in modern surgery, the difference between success and failure lies; he must be patient, too, in dealing with all the little obstacles which crop up. And he must have tact and a quick judgment of the idiosyncrasies of his patient, which he must be both quick to appreciate, and, within proper limits, to bend to. For the very nature of our work precludes the possibility of the patient being able to judge even of results, except by the test of time, far less of what is best to be done for him, so that the dentist has ample opportunity for the exercise of all his discretion in knowing when to give way to his patient, and when to fight out his little battle in the patient's own interest. And it is very desirable that he should cultivate a thoroughly kind and friendly feeling toward those who honor him with their confidence—I say cultivate, because I believe that such a habit of mind is strengthened by use, and that it is just as easy to entertain a friendly feeling towards those to whom we are able to render service, as it lies deep down in imperfect human nature to dislike those whom we have in any way injured. He must have a good physique; his work is hour after hour exhausting in a degree that no one who has not tried it can appreciate. With busy practice comes another difficulty, and that is to avoid being hurried, and to keep for each patient time enough to do him justice. There is no temptation for the busy dentist to spend one moment more than is absolutely necessary over his work; on the contrary, there is a very strong temptation in the other direction, as it becomes very difficult to satisfy all those who wish to be seen, and who do not realize that dental operations take so long that it is rarely possible for the dentist, as it sometimes may be for the medical man, to squeeze in another patient when his appointment book is full. So that a good deal of moral firmness is needed every day to keep the dentist out of this pitfall. And he has all the more need of these qualities in that his patient can never know the extent of the difficulties of his work—difficulties that are great enough, though the work be small—and will often be inclined to rate as high, or higher, the practitioner who attempts nothing difficult, but pilots their teeth towards a gradual and painless euthansia, as he who renders far more real service, but in attempting much more now and again fails in something that the other would never have attempted.

"It may be said that these qualities which I have sketched would have led to success in any calling; so I believe they would, and I fancy it is generally true that the man who scores a real success in any calling would have done so in a good many others had his career been a different one.

"One more word before I leave this matter of professional success. By success I do not mean merely pecuniary success. I do not call it real success unless a man stands in the opinion of his own professional brethren at least as high, or higher, than he does with the public. It is, unfortunately, the case that all the branches of the medical profession, and very especially in ours, the ear of the public is sometimes to be caught by self-assertion, and the many hydra-headed forms of quackery. It is sometimes asked why, when the manufacturer or the dealer advertises his goods without exciting the smallest adverse comment, should it be considered disgraceful for a barrister, a stock-broker, or a medical man to advertise himself. The difference is not far to seek, though it is often overlooked. The one advertises an article which he wishes to make known to the public, and it is greatly to their convenience that he should do so; the one extols a thing, the other extols a man—himself. And there is a further difference—the thing may be new, all that is said about it may be true, but this can hardly be the case with the personal advertisement. For all knowledge that is of importance in a professional sense is very soon public property, for each to make use of as his abilities serve; but it would hardly have the effect he desires were the advertiser to say: 'I am even as other men are;' he must brag in some form, or it would be no good, and when he brags he can hardly be truthful.

"Let us turn from this disagreeable subject to a consideration of the reaction upon the man himself of success in practice. Wealth he can hardly attain—the limits of time preclude it; and the great income of a surgeon or physician in the front rank is impossible. But ease and comfort and moderate savings are within the reach of a large number. He will have but little leisure; the large expenditure of time upon his operations in order to do them properly not merely sets a limit upon the amount that he can do, but the number of hours during which a man can do such work without undue exhaustion being soon reached, he has none too much energy for other things. One day's work very closely resembles the next, and the next, and though I would not be understood to say that there is not more of variety, and more scope for the exercise of sound judgment than any outsider might suppose, nevertheless, it is all exercised upon a strictly limited class of subjects, and so has its special mental dangers. The dentist in large practice may be compared to a man who daily journeys along a deep lane, shut in with hedgerows on either side. In such a lane there will be much for him who has eyes to see it, more, perhaps, than in a lifetime he can possibly exhaust, if he observes its geology, its fauna and flora, and the phenomena of human life and its ways that unfold themselves there; but for all that, our wayfarer will never understand even his little world if he never looks outside it. I came across a passage in one of Stevenson's novels the other day which illustrates what I mean: 'The dull man is made, not by the nature, but by the degree of his immersion in a single business'. And all the more if that be sedentary, uneventful, and ingloriously safe. More than one-hatf of him will then remain unexercised and undeveloped; the rest will be distended and deformed by over-nutrition, over-cerebration, and the heat of rooms.' And, inasmuch as it is easy to see the mote in our brother's eye, I often fancy that I can trace the cramping and narrowing effect of our necessarily limited horizons, which prevents our even seeing what is really well within their limits. There are countless problems lying before us; the etiology of the diseases we have to treat, problems of heredity laid out before us—a rich and varied field for observation, yet how many cultivate it, even making due allowance for the fatigues of our routine work. By all means, then, let the dentist who would keep his mind fresh cultivate a hobby. A hobby is more restful than idleness, and is a joy forever, if it be well chosen. I recollect being struck with the sadness of the end of the life of one of the greatest physicians of recent days, who had no hobbies. He broke down in health, so that he could not practice, and then time hung heavy, even on the hands of a bright intellect, because, with failing health and declining years, it was too late to take up a fresh pursuit. And, as a contrast, the end of the life of a great surgeon, who, when he retired from practice, eagerly turned to the pursuit of art, which he had cultivated with a great measure of success throughout a long and busy life. And I think I can trace the same cramping effect in our relations to outside matters.

"Important to the well-being of the individual are his teeth; yet man is not wholly a complex organism constructed for the purpose of carrying about thirty-two (or fewer) teeth. Useful as I hope we are, we are only a small section of a great community, and while we hope that any legislation which we may be able at any future time to influence, will be upon the lines, on which we have sought to improve the position of our profession, we must always remember that it is only because the advancement of our profession is, broadly speaking, for the public weal, that what has been effected in the past was possible, and that which may be effected in the future can come into the sphere of possibility only upon the same grounds of a general public utility."—*Journal of the British Dental Association.*

---

ARTICLE II.

